# Cytoflavin's neuroprotective effect in acute ischemic stroke: a systematic review and meta-analysis

**DOI:** 10.3389/fstro.2026.1775376

**Published:** 2026-10-30

**Authors:** Retnaningsih Retnaningsih, Dodik Tugasworo, Wati Arinta Puspita, Chandra Elvika, Arrizki Azka Pratama

**Affiliations:** Department of Neurology, Faculty of Medicine, University of Diponegoro, Semarang, Indonesia

**Keywords:** antioxidant therapy, Cytoflavin, ischemic stroke, meta-analysis, neuroprotection, NIHSS, systematic review

## Abstract

Acute ischemic stroke (AIS) remains a leading cause of long-term disability globally. While reperfusion therapies are effective, there is a growing need for adjunct neuroprotective strategies to minimize secondary brain injury. Cytoflavin, a metabolic and antioxidant compound, has shown potential neuroprotective effects. However, its clinical efficacy remains underexplored. This systematic review and meta-analysis aim to evaluate the efficacy of Cytoflavin in AIS, focusing on neurological recovery (NIHSS), functional independence (Barthel Index), cognitive performance (MMSE), and infarct volume. A systematic search was conducted across multiple databases for studies evaluating Cytoflavin in AIS. Nine studies were included, with a combined enrollment of 3,254 participants across treatment arms (sample sizes reported per arm); the number of unique participants contributing to each pooled outcome was smaller after correcting for shared control groups and repeated-measures overlap across evaluation time points (e.g., 1,379 for NIHSS). Data were analyzed using random-effects models; where a shared control group was compared against two Cytoflavin dose arms within the same study, the control group was split proportionally between the two comparisons to avoid double-counting participants (unit-of-analysis correction). Risk of bias was assessed using Cochrane Risk of Bias tool and Newcastle-Ottawa Scale. Cytoflavin significantly improved NIHSS scores (MD = −2.63, 95% CI: −3.15 to −2.10; moderate certainty) and Barthel Index (MD = 19.29, 95% CI: 9.52 to 29.07; moderate certainty), and showed improvements of low certainty in MMSE scores (MD = +6.93, 95% CI: 1.39 to 12.47) and mRS scores (MD = −0.41, 95% CI: −0.74 to −0.09), the latter derived from continuous summary statistics for an inherently ordinal scale (see Limitations). For infarct volume, effects diverged by evaluation time point: a marginal increase was observed at Day-10 (MD = +0.17 cm^3^, based on a single study), while a non-significant reduction was observed at Day-21 (MD = −4.73 cm^3^, 95% CI: −11.41 to 1.95; very low certainty). High heterogeneity (I^2^ ranging from 0% to 100%) was observed across outcomes and persisted after the unit-of-analysis correction, indicating genuine clinical and methodological diversity across studies. In conclusion, cytoflavin shows promising neuroprotective effects in AIS. However, due to heterogeneity and methodological limitations, further large-scale, high-quality studies are needed to confirm its efficacy and optimize treatment protocols.

**Systematic review registration:** https://www.crd.york.ac.uk/PROSPERO/view/CRD420251112430, identifier: CRD420251112430.

## 1 Introduction

Acute ischemic stroke (AIS) is a leading cause of mortality and long-term disability worldwide. It occurs due to a sudden obstruction of cerebral blood flow, most commonly from thromboembolism, resulting in a cascade of ischemic injury and neuronal death (Feigin et al., 2017). AIS accounts for approximately 85% of all stroke cases globally and contributes significantly to the global burden of neurological disease (Johnson et al., 2019). In Indonesia, stroke remains the leading cause of death and disability, with an age-standardized mortality rate of 193.3 per 100,000 population and significant economic burden on healthcare systems (Venketasubramanian et al., 2022).

Although recent advancements in reperfusion therapy, such as intravenous thrombolysis and mechanical thrombectomy, have improved outcomes for some patients, these interventions are time-sensitive and not universally applicable (Taha et al., 2021; Elwan et al., 2022; Kurnianto et al., 2021). Intravenous thrombolysis using recombinant tissue plasminogen activator (rtPA) remains the standard of care within 4.5 h of symptom onset, with an optimal door-to-needle time of less than 60 min to maximize outcomes (Elsayed et al., 2019). However, clinical audits in Indonesian stroke centers show that achieving these targets remains challenging, with mean DTN times exceeding 60 min and highlighting the need for system improvements (Retnaningsih and Hendartono, 2019). Strict inclusion criteria and limited access in many regions create substantial treatment gaps. Consequently, there is an urgent need to explore complementary neuroprotective strategies that can minimize ischemic brain injury and improve functional recovery for a broader patient population.

Oxidative stress biomarkers are thought to reflect not only acute injury in ischemic stroke but also longer-term outcomes such as cognitive impairment and post-stroke depression, reinforcing the rationale for neuroprotective strategies that extend beyond the acute phase (Tugasworo et al., 2023).

Cytoflavin, a metabolic compound comprising succinic acid, riboflavin, inosine, and nicotinamide, has been used extensively in Russia and surrounding regions as an adjunctive treatment for AIS. Its proposed mechanisms include enhancement of cellular energy metabolism, reduction of oxidative stress, and promotion of cerebral perfusion. Despite its regional popularity, Cytoflavin has not gained widespread international recognition, and robust evaluations of its clinical efficacy remain limited in the global literature (Skvortsova et al., 2010).

This systematic review and meta-analysis aims to evaluate the neuroprotective effects of Cytoflavin in patients with acute ischemic stroke. Specifically, it examines its impact on neurologic severity (NIHSS), functional recovery (Barthel Index, mRS), cognitive performance (MMSE), and infarct volume.

## 2 Method

### 2.1 Search strategy

The protocol for this systematic review and meta-analysis was prospectively registered in the International Prospective Register of Systematic Reviews (PROSPERO) under the registration number CRD420251112430. A systematic literature search was conducted across the following databases: PubMed, Embase, Scopus, ProQuest, ScienceDirect, Google Scholar, and the Cochrane Library. The search was conducted using the following terms and Boolean operators:

- “Cytoflavin” OR “Cytoflavine” OR (“succinic acid” AND “inosine” AND “nicotinamide” AND “riboflavin”)
- “Ischemic stroke” OR “acute ischemic stroke”
- “Neuroprotection” OR “neuroprotective effect”

Search strings were adapted to each database. For instance, the specific search string utilized in PubMed was as follows: (“Cytoflavin”[All Fields] OR “Cytoflavine”[All Fields] OR (“succinic acid”[All Fields] AND “inosine”[All Fields] AND “nicotinamide”[All Fields] AND “riboflavin”[All Fields])) AND (“Ischemic stroke”[All Fields] OR “acute ischemic stroke”[All Fields]) AND (“Neuroprotection”[All Fields] OR “neuroprotective effect”[All Fields]). Reference lists of included articles were also manually screened to identify any additional relevant studies (ancestry approach). The last search was conducted in April 2025. The search was deliberately focused on peer-reviewed bibliographic databases; clinical trial registries were not searched separately, as this review's inclusion criteria required published outcome data suitable for quantitative synthesis, which registry entries for ongoing or unpublished trials typically do not provide. In addition to the brand name, the search terms included the drug's constituent components (succinic acid, inosine, nicotinamide, and riboflavin, searched in combination) to capture studies that may describe the formulation by its ingredients rather than its trade name; a supplementary check of this combination against bibliographic and gray-literature sources identified no additional eligible primary studies within the original search window beyond those already captured under the Cytoflavin/Cytoflavine terms.

For full transparency and reproducibility, the complete adapted search string used for each of the seven databases searched was adapted from the PubMed strategy shown above. PubMed, Embase, Scopus, ProQuest, and the Cochrane Library support the full nested Boolean logic shown; ScienceDirect and Google Scholar have more limited advanced-search syntax and do not support deeply nested parenthetical logic, so a simplified equivalent was used for these two databases, which may have modestly reduced sensitivity to the ingredient-combination branch of the search relative to the other five databases.

### 2.2 Inclusion and exclusion criteria

Study eligibility was defined in advance using the PICOS framework (Population, Intervention, Comparator, Outcomes, Study Design; detailed in Section 2.3, Data Extraction and Eligibility Framework), which forms the basis of the following inclusion and exclusion criteria.

#### 2.2.1 Inclusion criteria:

- Randomized controlled trials (RCTs), observational study, or prospective clinical trials.
- Studies involving patients diagnosed with acute ischemic stroke (AIS).
- Studies that evaluated the neurological or functional outcomes of Cytoflavin administration.
- Articles published in English or Russian.

#### 2.2.2 Exclusion criteria:

- Preclinical studies, animal or in vitro studies.
- Reviews, editorials, conference abstracts, or case reports.
- Studies involving primary hemorrhagic stroke or chronic-phase rehabilitation as their main population or focus.
- Non-English or non-Russian articles without available full text or translation.

### 2.3 Data extraction and eligibility framework (PICOS)

Two independent reviewers extracted data from each included study using a standardized form. The eligibility criteria were structured using the PICOS framework:

- **Population (P):** Adult patients diagnosed with acute ischemic stroke (AIS).
- **Intervention (I):** Administration of Cytoflavin (succinic acid, riboflavin, inosine, and nicotinamide), given intravenously and/or orally, as an adjunct to standard stroke therapy.
- **Comparator (C):** Placebo, standard stroke therapy without Cytoflavin, or an alternative neuroprotective/metabolic regimen.
- **Outcomes (O):** Neurological severity (NIHSS), functional recovery (Barthel Index, modified Rankin Scale), cognitive performance (MMSE), and infarct volume.
- **Study design (S):** Randomized controlled trials (RCTs) or observational (cohort/comparative) studies with a comparator group, as detailed in the inclusion and exclusion criteria above (Section 2.2).

Regarding statistical data types, for continuous outcomes like NIHSS, MMSE, and Barthel, data were extracted as Mean ± SD. For outcomes reported using Median ± IQR, data were extracted as reported, with the necessary transformations described in the Data Synthesis section. Disagreements between reviewers were resolved through discussion or by consultation with a third reviewer.

### 2.4 Risk of bias and quality assessment

Risk of bias (RoB) was assessed using the Cochrane Risk of Bias 2.0 (RoB 2) tool for randomized controlled trials (RCTs) and the Newcastle-Ottawa Scale (NOS) for observational studies.

The Newcastle-Ottawa Scale (NOS) was selected over the more recently developed ROBINS-I tool for appraising the observational studies in this review. Although ROBINS-I offers a more granular, domain-by-domain assessment of confounding and is increasingly recommended for comparative effectiveness reviews, its proper application requires a prespecified list of confounders and detailed reporting of how each was measured and adjusted for, information that was not consistently reported in sufficient granularity across the included observational studies, several of which were single-center reports published in Russian-language clinical journals with more limited methodological detail than typically expected by ROBINS-I. The NOS, by contrast, is a well-established and widely applied tool for appraising cohort and comparative observational studies of this kind, and could be applied consistently across all included observational studies using the information they reported. We acknowledge this as a methodological limitation (see Strengths and Limitations) and note that a future update of this review, or reanalysis by independent investigators with access to fuller study-level confounder data, may benefit from applying ROBINS-I instead.

- RoB 2 evaluates five key domains: randomization process, deviations from intended interventions, missing outcome data, measurement of outcomes, and selection of reported results. Based on these domains, studies were classified as Low Risk, Some Concerns, or High Risk (Sterne et al., 2019; Lo et al., 2014).
- NOS evaluates three broad categories for observational (cohort/comparative) studies: Selection (4 items, maximum 4 points), Comparability (1 item, maximum 2 points, with partial credit of 0.5 points available when a study adjusts for some but not all prespecified confounders), and Outcome Assessment (3 items, maximum 3 points), for a maximum total score of 9 points. Consistent with a widely used convention for applying the NOS, studies were classified as High Quality (7-9 points), Moderate Quality (4-6 points), or Low Quality (0-3 points) based on their total NOS score.

These classifications helped determine the reliability of the studies included in the meta-analysis.

### 2.5 Data synthesis

For the meta-analysis, the data from the included studies were first standardized to ensure consistency across studies. Continuous data, such as NIHSS, Barthel Index, MMSE, and Volume Infarct, were all expressed as Mean ± Standard Deviation (SD). For studies that reported Median and Interquartile Range (IQR), the median and IQR were converted to Mean ± SD using the method described by Wan et al. (2014). This process ensures that data from different studies could be pooled appropriately and validly.

In two included studies, Rumyantseva et al. (2017) and Sazonov et al. (2016), Cytoflavin was evaluated at two doses (10 mL/day and 20 mL/day) against a single shared control group within the same trial. Treating each dose-arm-vs.-control comparison as an independent study in the pooled analysis would count participants in the shared control arm twice, artificially inflating the total sample size and precision of the pooled estimate, a unit-of-analysis error as described in the Cochrane Handbook for Systematic Reviews of Interventions (Section 6.5.2.10) (Higgins et al., 2023). To correct for this, the shared control group in each of these two studies was split proportionally between the two dose-arm comparisons, in proportion to the sample size of each corresponding intervention arm, while the reported mean and standard deviation of the control group were kept unchanged for both comparisons. This approach preserves each dose arm as a separate comparison against control (an analytic choice retained because it enables examination of a potential dose-response relationship; see Sazonov et al., 2017, and Discussion), while avoiding double-counting of shared control participants across the pooled estimate.

To characterize the time-course of the treatment effect, subgroup analysis was conducted by dividing the data into two evaluation time points: Day-10 and Day-21. This approach allowed us to examine the treatment effect separately at each time point, providing a more detailed picture of Cytoflavin's effects over the course of recovery.

Random-effects models were used for all outcomes, as heterogeneity across studies was expected. The Mean Difference (MD) was calculated for each continuous outcome at both time points. Pooled effect sizes were calculated with 95% Confidence Intervals (CI) to evaluate the treatment effects at both Day-10 and Day-21, giving an overall estimate of Cytoflavin's impact across the evaluation periods.

Forest plots were generated to visually display the pooled effects for both time points. These plots illustrate the treatment effects and confidence intervals for each outcome, allowing for comparison between the two evaluation periods and providing a more complete view of the treatment's overall effect. To evaluate the robustness of our pooled estimates and minimize the risk of confounding bias, a sensitivity analysis was performed by separately assessing randomized controlled trials (RCTs) and excluding observational studies. Statistical heterogeneity across studies was assessed using the I^2^ statistic, the Q statistic, and Tau^2^, with I^2^ values of approximately 25%, 50%, and 75% interpreted as indicating low, moderate, and high heterogeneity, respectively. Publication bias was evaluated using funnel plots complemented by Egger's and Begg's tests for outcomes with a sufficient number of included studies (Sterne et al., 2011). The overall certainty of evidence for each outcome was assessed using the Grading of Recommendations Assessment, Development and Evaluation (GRADE) framework, considering risk of bias, inconsistency, indirectness, imprecision, and publication bias. Meta-regression to explore potential sources of heterogeneity (e.g., Cytoflavin dosage or baseline NIHSS severity) was prespecified as a planned analysis contingent on a minimum of 10 studies per covariate, consistent with the threshold recommended in the Cochrane Handbook for Systematic Reviews of Interventions (Higgins et al., 2023, Section 10.11) for a sufficiently powered and reliable model; given the limited number of trials available per clinical outcome, this threshold was not met for any outcome, and meta-regression was therefore not performed.

## 3 Theory

Acute ischemic stroke (AIS) initiates a complex cascade of pathophysiological events including oxidative stress, mitochondrial dysfunction, excitotoxicity, inflammation, and apoptosis. These processes not only cause primary injury but also contribute to delayed neuronal damage in the ischemic penumbra. While reperfusion therapies such as intravenous thrombolysis and mechanical thrombectomy are effective in restoring blood flow, they do not directly target cellular injury mechanisms and can even exacerbate injury through reperfusion-related oxidative stress and inflammation (Fisher, 2023).

Cytoprotection offers a complementary approach aimed at preserving the structure and function of neurons and glial cells. Unlike neuroprotection that narrowly focuses on neuronal survival, cytoprotection involves multi-target strategies that stabilize mitochondrial energy metabolism, counteract oxidative injury, and modulate neuroinflammation (Shehadah et al., 2011; Gulyás et al., 2012).

Cytoflavin, a pharmacological compound containing succinic acid, riboflavin (vitamin B2), inosine, and nicotinamide, exerts cytoprotective effects through multiple mechanisms. Succinic acid facilitates ATP production through the Krebs cycle; riboflavin supports antioxidant enzyme systems; inosine provides neurotrophic and anti-inflammatory functions; and nicotinamide supports DNA repair and NAD+ metabolism. Together, these components enhance mitochondrial function, reduce reactive oxygen species (ROS), and promote neuronal resilience in the face of ischemic injury (Poltavskaya et al., 2020; Kataeva et al., 2022). Recent studies support these mechanisms. For example, Cytoflavin has been shown to improve MMSE and NIHSS scores in patients during early recovery after AIS (Poltavskaya et al., 2020; Karakulova et al., 2018) further demonstrated Cytoflavin's role in increasing neurotrophin levels and improving cognitive recovery, suggesting modulation of neuroplasticity (Karakulova et al., 2018).

Additionally, elevated inflammatory biomarkers such as neutrophil-to-lymphocyte ratio (NLR) and platelet-to-lymphocyte ratio (PLR) have been associated with worse stroke outcomes (Retnaningsih et al., 2023). Cytoflavin's ability to modulate inflammatory responses could potentially mitigate such risks. This aligns with data showing that systemic inflammation persists for days after stroke and is closely tied to microglial activation in peri-infarct zones (Gulyás et al., 2012). Further, beyond innate immune responses, adaptive immunity also plays a critical role in the progression and resolution of post-stroke inflammation. A notable study by Frenkel et al. demonstrated that CD4? T-cells producing interleukin-10 (IL-10) conferred neuroprotection following ischemic stroke, reducing infarct size and improving outcomes in murine models (Frenkel et al., 2005). This highlights the therapeutic potential of modulating anti-inflammatory T-cell responses to limit secondary neuronal damage. Although Cytoflavin is not directly an immunomodulator, its capacity to reduce oxidative and metabolic stress may indirectly promote a favorable immune microenvironment, allowing beneficial immune responses such as IL-10-mediated mechanisms to predominate.

Cerebral ischemia also leads to glutamate accumulation and excitotoxicity, contributing to intracellular calcium overload and neuronal apoptosis. This process is particularly intense during reperfusion, amplifying oxidative stress and mitochondrial damage (Chamorro et al., 2016). Cytoflavin's antioxidant and metabolic support may counteract these excitotoxic mechanisms, offering protection in the critical early phase. Moreover, ischemic stroke disrupts the blood-brain barrier (BBB) and neurovascular unit (NVU), exacerbating inflammation and edema. Agents that restore mitochondrial function and reduce oxidative stress can preserve BBB integrity (Abdullahi et al., 2018). Components of Cytoflavin, particularly nicotinamide and succinate, may strengthen endothelial resilience and limit secondary injury. Biomarkers such as MDA (malondialdehyde), 8-OHdG (8-hydroxy-2′-deoxyguanosine), and cytokine panels (e.g., IL-6, TNF-α) have been proposed to track oxidative and inflammatory damage. Imaging tools like diffusion-weighted imaging (DWI) and perfusion-weighted imaging (PWI) also provide early indications of salvageable penumbra, where cytoprotection is most effective (Tugasworo et al., 2023).

A study by Shehadah et al. highlights the advantage of combining a metabolic agent (Niaspan) with tPA, which showed reduced infarct volume and improved outcomes compared to either agent alone (Shehadah et al., 2011). This supports the concept of integrating cytoprotection with reperfusion therapy. While thrombolytic therapy has a narrow time window (typically < 4.5 h) (Hacke et al., 2008; Tsivgoulis et al., 2023), cytoprotective agents may retain efficacy beyond this window, providing therapeutic flexibility (Saver, 2006). Comparative studies show that Cytoflavin's multifactorial profile may offer broader benefits than single-pathway agents such as edaravone (free radical scavenger) or citicoline (membrane stabilizer). This broad mechanism also makes Cytoflavin suitable for combination with reperfusion and rehabilitation strategies.

The conceptual framework of cytoprotection is further reinforced by emerging understanding of post-stroke molecular dynamics. As noted by Fisher, revisiting cytoprotective therapies is essential in the reperfusion era, especially for agents with demonstrated preclinical efficacy and acceptable safety profiles (Fisher, 2023). Collectively, these findings support the integration of cytoprotective agents like Cytoflavin into AIS treatment protocols as adjuvants to reperfusion therapy. Their multi-targeted mechanisms are particularly valuable in mitigating the cellular aftermath of ischemia and reperfusion, potentially enhancing long-term functional and cognitive outcomes.

## 4 Results

### 4.1 Study selection

Study selection and reporting followed the PRISMA 2020 guidelines (Page et al., 2021). A total of 104 records were identified: 92 through database searches (PubMed *n* = 5, Embase *n* = 0, Scopus *n* = 16, ProQuest *n* = 0, ScienceDirect *n* = 0, Cochrane Library *n* = 0, and Google Scholar *n* = 71) and 12 through manual screening of reference lists of the included studies (ancestry approach). After removing 5 duplicates, 99 records were screened for eligibility.

Following this initial screening, 83 records were excluded due to irrelevance to the review objective or failure to meet inclusion criteria based on title and abstract content. 16 full-text articles were retrieved and assessed for eligibility.

Following full-text review, 7 studies were excluded: 5 for evaluating a subacute rather than acute stroke phase, 1 for evaluating a chronic stroke phase, and 1 (Majidova and Khalilova, 2020) for enrolling a predominantly pediatric population (mean age 9.14 +/– 5.6 years) and using pediatric-specific outcome measures (pedNIHSS, PSOM-SNE), which violated this review's PICOS eligibility criterion restricting inclusion to adult patients with acute ischemic stroke, as detailed in the PRISMA flowchart. Nine studies met the eligibility criteria and were included in the final quantitative synthesis. The complete study selection process is illustrated in the PRISMA flowchart (Figure 1).

**Figure 1:**
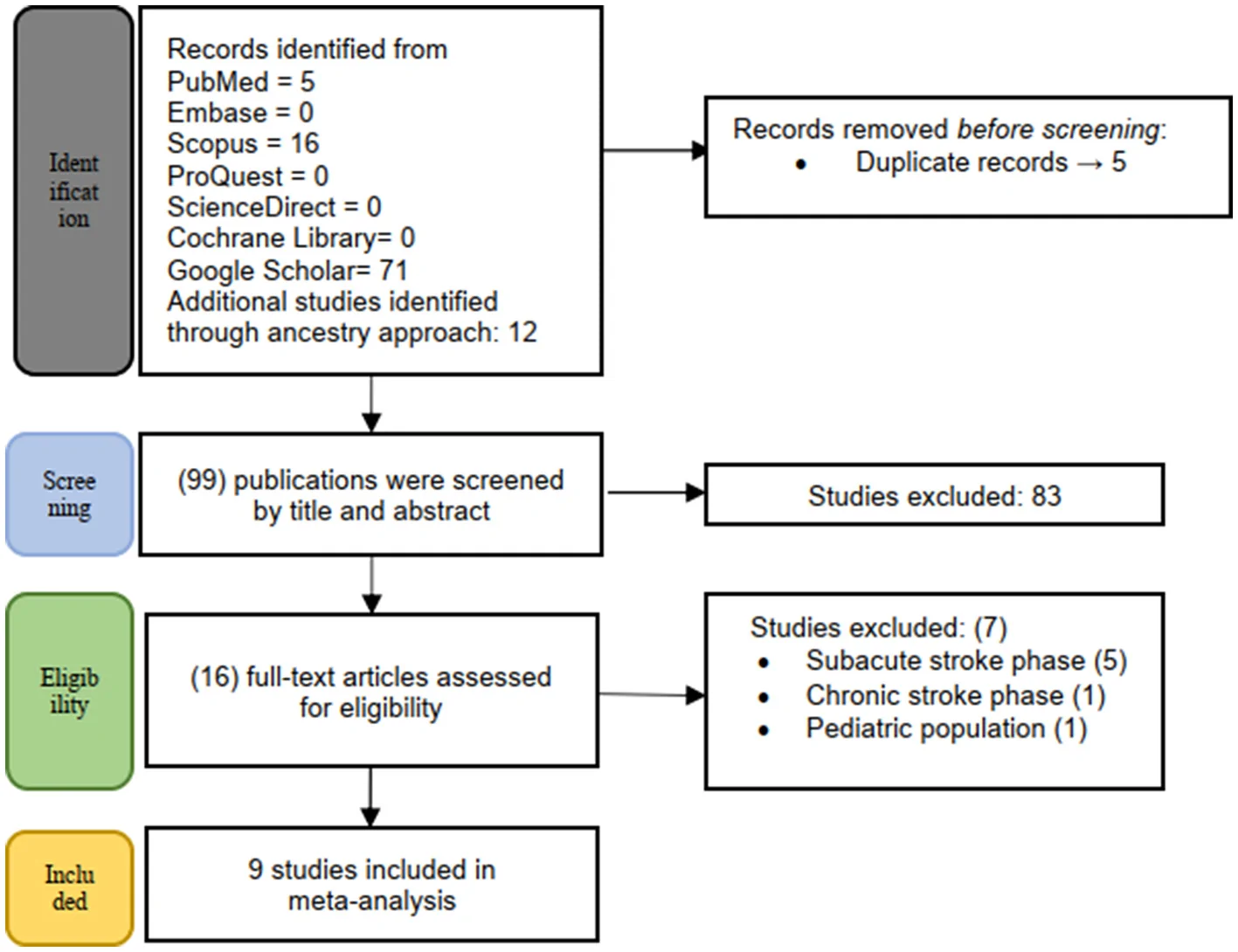
PRISMA flowchart.

### 4.2 Study characteristics

The 9 included studies were published between 2003 and 2023 and were conducted in Russia and Uzbekistan. These studies included randomized controlled trials (RCTs) and observational studies, including cohort studies. The sample sizes ranged from 26 to 725 participants (per treatment arm; see Table 1). Reported mean or median participant age ranged from 57.2 to 73.9 years across the seven studies providing a single summary age value; two further studies (Sazonov et al., 2016; Fedin et al., 2005) reported only the overall age range of their enrolled populations (43–85 years and 45–60 years, respectively) rather than a mean or median. All studies assessed ischemic stroke and the effects of Cytoflavin as an adjunct to standard stroke therapies, such as thrombolysis or thrombectomy.

**Table 1:** Characteristics of the 9 included studies.

No	First Author, Year	Country	Study design	N (Cytoflavin)	N (Control)	Average age (Cytoflavin/Control)	Therapy setting	Cytoflavin dosage & duration	Additional therapy	Evaluation time	Outcome measure	Risk of Bias
1.	Muratov et al., 2016	Uzbekistan & Russia	RCT	60	60	61,0 ± 4,9/62,2 ± 5,3	Combination	IV: 20 ml, 2x/day (day 1-10) 2 tablet PO 2x/day (day 11-35)	Standard therapy	Day 1, 10, 35	NIHSS, MMSE, Barthel, mRS, Infarct volume	Some concerns
2.	Sazonov et al., 2017	Russia	RCT	Dose 10ml: 27 Dose 20 ml: 29	30	43–85 (range)	Combination	IV 10 ml /day (10 days) or IV 20 ml/day (10 days)	Standard therapy	Day 1, 5, 10	NIHSS, Barthel, mRS, Rivermead Mobility Index	Some concerns
3.	Sazonov et al., 2016	Russia	Observational	29	26	73,9/69,3	Combination	IV 10 ml (10 days)	Thrombolytic & neuroprotective therapy	Pre-therapy, 1hr, 1 day	NIHSS, mRS, Barthel	High Quality (8/9)
4.	Polysan, 2003	Russia	RCT	150	150	57.2 ± 10.8	Combination	IV 10 ml (10 days)	Antiplatelets & antihypertension (if needed)	Day 11, Day 30	MMSE, Tinetti, Barthel, EEG	Some concerns
5.	Fedin et al., 2005	Russia	RCT	300	300	45–60 (range)	Combination	IV 400 ml (2x/day)	Background therapy	Day 21, Day 120	NIHSS, Barthel, MMSE	Some concerns
6.	Rumyantseva et al., 2017	Russia	RCT	Cyt-10: 133 Cyt-20: 108	AA: 132	Cyt-10: 64.3 ± 8.2 Cyt-20: 64.4 ± 8.1 Control: 64.9 ± 8.6	Combination	IV 20 ml/day Cyt-10: 10 days Cyt-20: 20 days	Standard therapy	Day 1, 10, 21	NIHSS, Barthel, mRS, MMSE, Infarct volume, Rivermead Mobility Index	Some concerns
7.	Kovalenko et al., 2023	Russia	Observational	100	100	68 [58,75–74] (median) [IQR]	Combination	IV 10-20 ml (10 days)	Reperfusion therapy	Day 1, 2, 7, 10, 21,90	NIHSS, Barthel, mRS, Rivermead Mobility Index	High Quality (9/9)
8.	Skvortsova et al., 2010	Russia	RCT	41	29	64.4 ± 9.0/66.6 ± 9.8	Combination	20 ml IV (2x/day), tablets 850mg (2x/day)	Standard therapy	Day 1, 35-37	NIHSS, Barthel, Rankin, MMSE, Infarct volume	Some concerns
9.	Kovalchuk et al., 2018	Russia & Uzbekistan	Observational	725	725	69,7/67,3	Combination	10 ml IV (15 days), oral 2 tablets (2x/day for 2 months)	Neuropsychological therapy	Up to 7 months	MMSE, Depression Scale	High Quality (8/9)

Risk of Bias: for randomized controlled trials, the value shown is the overall risk-of-bias judgment from the Cochrane RoB 2 tool (see Table 2 for domain-level detail); for observational studies, the value shown is the Newcastle-Ottawa Scale (NOS) total score and quality band (see Table 3 for domain-level detail).

The outcomes included neurological severity (NIHSS), functional recovery (Barthel Index and mRS), cognitive improvement (MMSE), and infarct volume (measured by CT/MRI). The duration of Cytoflavin treatment ranged from 10 days to ≥21 days, with different administration routes (intravenous, oral). The comparator group received either placebo or standard therapy. Detailed study characteristics are presented in Table 1.

### 4.3 Quality of included studies

Risk of bias was evaluated for the randomized controlled trials (RCTs) and observational studies using the Cochrane Risk of Bias 2.0 (RoB 2) tool and the Newcastle-Ottawa Scale (NOS).

The RCTs in this review generally showed some concerns in several domains, particularly in randomization, measurement of outcomes, and deviations from intended interventions. The overall risk of bias was classified as Some Concerns for most RCTs, meaning that while these studies had valid designs, certain methodological issues may have affected their reliability. Detailed risk of bias for Randomized Controlled Trials are presented in Table 2.

**Table 2:** Quality assessment of the 6 included RCT studies.

Study	Randomization	Deviations from intended interventions	Missing outcome data	Measurement of outcome	Selection of reported results	Overall risk
Muratov et al., 2016	Some concerns	Some concerns	Low risk	Some concerns	Some concerns	Some concerns
Sazonov et al., 2016	Some concerns	Some concerns	Low risk	Some concerns	Some concerns	Some concerns
Polysan, 2003	Low risk	Some concerns	Low risk	Some concerns	Some concerns	Some concerns
Fedin et al., 2005	Low risk	Low risk	Low risk	Some concerns	Some concerns	Some concerns
Rumyantseva et al., 2017	Low risk	Some concerns	Low risk	Some concerns	Some concerns	Some concerns
Skvortsova et al., 2010	Some concerns	Low risk	Low risk	Some concerns	Some concerns	Some concerns

The three observational studies retained after full-text screening (Kovalenko et al., 2023; Sazonov et al., 2016; Kovalchuk et al., 2018) were all rated as High Quality according to the Newcastle-Ottawa Scale (NOS). Detailed risk of bias for these observational studies is presented in Table 3. Of these, Kovalchuk et al. (2018) did not contribute a row to any pooled forest plot, as its reported outcome data were not in a format compatible with mean-difference pooling (see Strengths and Limitations).

**Table 3:** Quality assessment of 3 included Observational Studies.

Study	Selection	Comparability	Outcome	Interpretation
Kovalenko et al., 2023	4/4	2/2	3/3	High Quality (9/9)
Sazonov et al., 2016	4/4	1/2	3/3	High Quality (8/9)
Kovalchuk et al., 2018	4/4	2/2	2/3	High Quality (8/9)

This review's conduct was also considered against the AMSTAR-2 critical appraisal tool. Several core elements were satisfied, including a priori PICOS criteria, PROSPERO registration, duplicate data extraction, risk-of-bias assessment using tools matched to each study design (RoB 2 for RCTs, NOS for observational studies), a random-effects model appropriate to the anticipated heterogeneity, and assessment of publication bias with explicit power caveats. Other elements, such as a fuller account of protocol adherence and a complete supplementary listing of full-text exclusions with reasons, represent opportunities for strengthening in a future update of this review.

### 4.4 Quantitative results

#### 4.4.1 NIHSS (national institutes of health stroke scale)

A total of 5 studies (10 dose-arm/timepoint comparisons across Day-10 and Day-21) were included in the meta-analysis for NIHSS (Table 1 lists a seventh study, Skvortsova et al. (2010), as also reporting NIHSS, but its extracted data were not in a mean-difference-compatible format and it does not contribute to the pooled analysis; see Strengths and Limitations). Sazonov et al. (2016), although reporting NIHSS, was not evaluated at either Day-10 or Day-21 (its evaluation timepoints were pre-therapy, 1 h, and 1 day post-therapy) and therefore did not contribute to either subgroup. Two studies (Rumyantseva et al., 2017 and Sazonov et al., 2016) contributed two Cytoflavin dose arms (10 mL/day and 20 mL/day) each compared against a single shared control group; to avoid double-counting shared control participants across dose-specific comparisons (unit-of-analysis error, Cochrane Handbook Section 6.5.2.10), the control group in each of these studies was split proportionally between the two dose-arm comparisons before pooling. After this correction, 779 participant-comparisons were included for Day-10 and 1,173 for Day-21. Because Kovalenko et al. (2023) and Rumyantseva et al. (2017) were evaluated at both time points, their participants are counted in both subgroups; the true number of unique participants contributing to the NIHSS outcome overall is 1,379 (779 + 1,173, less the 573 participants from these two studies who are counted in both subgroups), not the simple sum of 1,952 (itself already corrected from a previously reported, further double-counted total of 2,299 that also included the shared-control unit-of-analysis error). Outcome-specific numbers of unique participants are summarized in Table 4. Subgroup analysis was performed to assess the effects of Cytoflavin at both Day-10 and Day-21 evaluations.

**Table 4:** Summary of findings.

Outcome	Number of studies	Participants	Statistical method	Effect estimate	Publication bias	Certainty of evidence	Reasons for downgrading
NIHSS Improvement	5 studies (10 comparisons)	1379	Mean Difference (IV, Random, 95% CI)	−2.63 [−3.15, −2.10]	Egger *P* = 0.016, Begg *P* = 0.146 (k=10 comparisons across Day-10/Day-21; below ~10-study threshold for reliable interpretation)	Moderate	Substantial heterogeneity; possible small-study/publication-bias effect (see Egger's test)
Day-10	4 studies (6 comparisons)	779		−2.62 [−3.35, −1.88]			
Day-21	3 studies (4 comparisons)	1173		−3.32 [−6.53, −0.11]			
Barthel Index Improvement	5 studies (9 comparisons)	834	Mean Difference (IV, Random, 95% CI)	19.29 [9.52, 29.07]	Egger *P* = 0.70, Begg *P* = 0.92 (k=9 comparisons across Day-10/Day-21; below ~10-study threshold for reliable interpretation)	Moderate	Inconsistency (heterogeneity) across studies
Day-10	4 studies (6 comparisons)	634		14.42 [2.57, 26.26]			
Day-21	2 studies (3 comparisons)	573		29.54 [2.09, 56.98]			
MMSE Improvement	3 studies (6 comparisons)	1093	Mean Difference (IV, Random, 95% CI)	6.93 [1.39, 12.47]	Egger *P* = 0.94, Begg *P* = 0.72 (k=6; below ~10-study threshold for reliable interpretation)	Low	Risk of bias; inconsistency across studies
Day-10	2 studies (3 comparisons)	493		3.53 [1.83, 5.23]			
Day-21	2 studies (3 comparisons)	973		10.23 [4.44, 16.02]		Low	
Modified Rankin Scale (mRS), Day-10	3 studies (4 comparisons)	261	Mean Difference (IV, Random, 95% CI)	−0.41 [−0.74, −0.09]	Egger *P* = 0.97, Begg *P* = 1.00 (k=4; below ~10-study threshold for reliable interpretation)	Low	Risk of bias; inconsistency across studies
Infarct volume reduction	2 studies (3 comparisons)	493	Mean Difference (IV, Random, 95% CI)	See Day-10 and Day-21 below	Egger *P* = 0.392, Begg *P* = 0.543 (Day-21 subgroup, k=2; not computable for the single-study Day-10 subgroup; interpret with caution given the very small number of studies)	Very Low	Risk of bias; inconsistency; imprecision; potential publication bias
Day-10	1 study	120		0.17 [0.12, 0.22] cm^3^			
Day-21	1 study (2 comparisons)	373		−4.73 [-11.41, 1.95] cm^3^			

For Day-10, the pooled mean difference (MD) was −2.62 (95% CI: −3.35 to −1.88), favoring Cytoflavin over control in improving NIHSS scores. Heterogeneity for Day-10 was high (I^2^ = 98.6%), indicating considerable variability across the studies. The overall test for effect was significant (*P* < 0.00001).

For Day-21, the pooled MD was −3.32 (95% CI:−6.53 to −0.11), favoring Cytoflavin over control; after correcting the shared-control double-count, this effect reached statistical significance (*P* = 0.04), in contrast to the borderline result reported prior to this correction. Heterogeneity for Day-21 remained high (I^2^ = 98.1%).

The total pooled effect across both time points (Day-10 and Day-21) was −2.63 (95% CI: −3.15 to −2.10), showing a significant reduction in NIHSS scores favoring Cytoflavin over control, based on 1,379 unique participants (see Study Characteristics above for the repeated-measures adjustment). The overall heterogeneity for all studies combined remained high (I^2^ = 98.7%).

The data for NIHSS were synthesized using random-effects models, with the results visually represented in the forest plot, as shown in Figure 2, which illustrates the pooled effects of Cytoflavin on neurological function.

**Figure 2:**
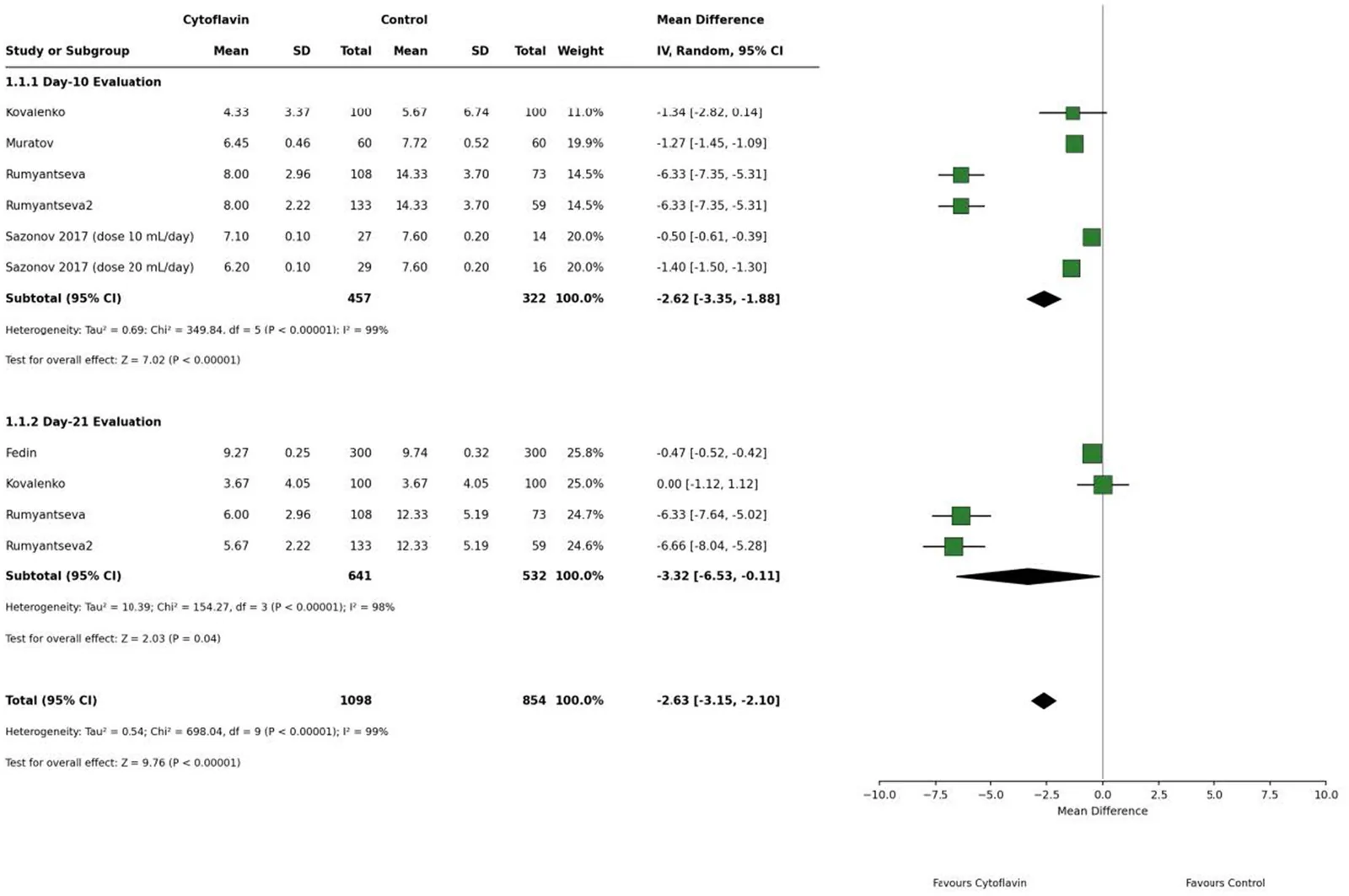
Forest plot of the meta-analysis assessing the effect of Cytoflavin vs. control on NIHSS outcomes at Day-10 and Day-21. The size of each square represents the weight of the study in the random-effects model, with horizontal lines showing the 95% confidence intervals. The diamond at the bottom indicates the pooled effect estimate and its 95% CI. The analyzed values reflect the post-treatment scores at the respective evaluation time points.

Publication bias was re-verified using Egger's and Begg's tests, computed directly from the 10 study-level comparisons (across both Day-10 and Day-21) underlying the split-control-corrected dataset described above. Egger's test (*P* = 0.016) indicated a possible small-study/publication-bias effect, while Begg's test (*P* = 0.146) did not; taken together with the limited number of comparisons available (see Limitations), this result should be interpreted cautiously rather than as definitive evidence of publication bias. According to the GRADE assessment, the certainty of evidence was rated as moderate, downgraded due to substantial heterogeneity and possible small-study effects.

#### 4.4.2 Barthel index

A total of 5 studies were included in the meta-analysis for Barthel Index (Table 1 lists three additional studies, Fedin et al. (2005), Skvortsova et al. (2010), and Polysan (2003), as also reporting this outcome, but their data do not contribute a row to the pooled analysis; see Strengths and Limitations). As with NIHSS, the shared control group in Rumyantseva et al. (2017) and Sazonov et al. (2016) was split proportionally between the two Cytoflavin dose-arm comparisons within each study to correct for the unit-of-analysis error described above (Cochrane Handbook Section 6.5.2.10). After this correction, 634 participant-comparisons were included for Day-10 and 573 for Day-21. Because Rumyantseva et al. (2017) was evaluated at both time points, its 373 participants are counted in both subgroups; the true number of unique participants contributing to the Barthel Index outcome overall is 834 (634 + 573, less this 373-participant overlap), not the simple sum of 1,207. Subgroup analysis was performed to assess the effects of Cytoflavin at both Day-10 and Day-21 evaluations.

For Day-10, the pooled mean difference (MD) was 14.42 (95% CI: 2.57 to 26.26), favoring Cytoflavin over control in improving functional independence. Heterogeneity for Day-10 remained high (I^2^ = 100%), indicating substantial variability across the studies. The overall test for effect was significant (*P* = 0.02).

For Day-21, the pooled MD was 29.54 (95% CI: 2.09 to 56.98), favoring Cytoflavin over control in improving functional independence; this effect remained statistically significant after correcting the shared-control double-count (*P* = 0.03), consistent with the result reported prior to this correction. Heterogeneity for Day-21 remained high (I^2^ = 97.6%).

The total pooled effect across both time points (Day-10 and Day-21) was 19.29 (95% CI: 9.52 to 29.07), showing a significant improvement in Barthel Index scores favoring Cytoflavin over control, based on 834 unique participants (see Barthel Index paragraph above for the repeated-measures adjustment). The overall heterogeneity for all studies combined remained high (I^2^ = 100%). The data for Barthel Index were synthesized using random-effects models, with the results visually represented in the forest plot, as shown in Figure 3, which illustrates the pooled effects of Cytoflavin on functional independence.

**Figure 3:**
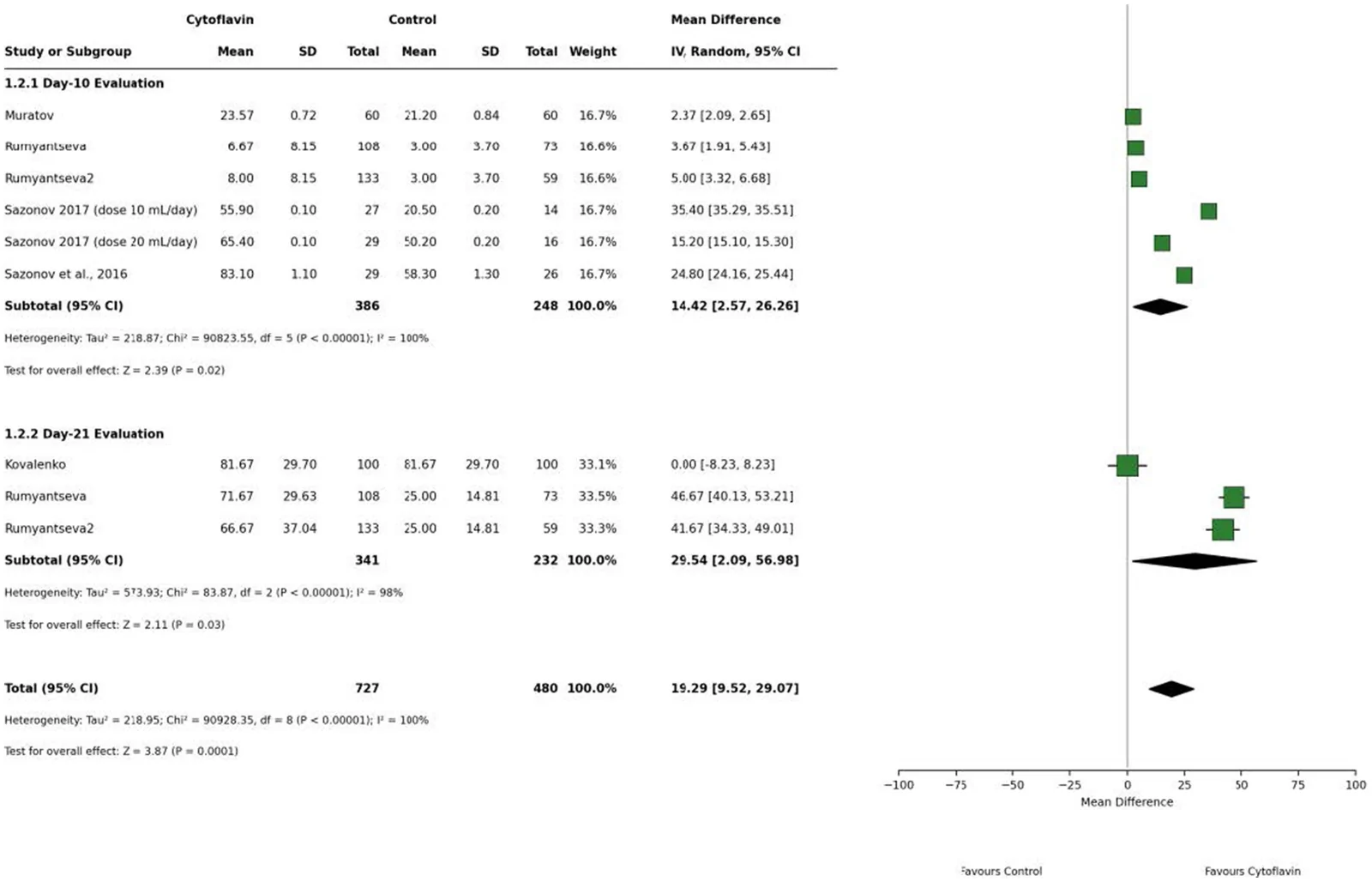
Forest plot of the meta-analysis assessing the effect of Cytoflavin vs. control on Barthel Index at Day-10 and Day-21. The size of each square represents the weight of the study in the random-effects model, with horizontal lines showing the 95% confidence intervals. The diamond at the bottom indicates the pooled effect estimate and its 95% CI. The analyzed values reflect the post-treatment scores at the respective evaluation time points.

Publication bias was re-verified using Egger's and Begg's tests, computed directly from the 9 study-level comparisons (across both Day-10 and Day-21) underlying the split-control-corrected dataset. Egger's test (*P* = 0.70) and Begg's test (*P* = 0.92) both indicated no significant publication bias. According to the GRADE assessment, the certainty of evidence was rated as moderate, mainly downgraded due to inconsistency across studies.

#### 4.4.3 MMSE (Mini-Mental State Examination)

A total of 3 studies (6 dose-arm/timepoint comparisons across Day-10 and Day-21) were included in the meta-analysis for MMSE. As with NIHSS and Barthel Index, the shared control group in Rumyantseva et al. (2017) was split proportionally between the two Cytoflavin dose-arm comparisons to correct for the unit-of-analysis error described above. After this correction, 493 participant-comparisons were included for Day-10 and 973 for Day-21. Because Rumyantseva et al. (2017) was evaluated at both time points, its 373 participants are counted in both subgroups; the true number of unique participants contributing to the MMSE outcome overall is 1,093 (493 + 973, less this 373-participant overlap), not the simple sum of 1,466. Subgroup analysis was performed to assess the effects of Cytoflavin at both Day-10 and Day-21 evaluations.

For Day-10, the pooled mean difference (MD) was 3.53 (95% CI: 1.83 to 5.23), favoring Cytoflavin over control in improving cognitive function. Heterogeneity for Day-10 remained high (I^2^ = 81.9%), indicating considerable variability across the studies. The overall test for effect was significant (*P* < 0.0001).

For Day-21, the pooled MD was 10.23 (95% CI: 4.44 to 16.02), showing a stronger improvement in MMSE scores favoring Cytoflavin at this later time point. Heterogeneity for Day-21 remained high (I^2^ = 97.4%), with the test for overall effect yielding *P* = 0.0005, indicating a significant effect at this later evaluation point.

The total pooled effect across both time points (Day-10 and Day-21) was 6.93 (95% CI: 1.39 to 12.47), demonstrating a significant improvement in MMSE scores favoring Cytoflavin over control, based on 1,093 unique participants (see MMSE paragraph above for the repeated-measures adjustment). The overall heterogeneity for all studies combined remained very high (I^2^ = 99.8%). The data for MMSE were synthesized using random-effects models, with the results visually represented in the forest plot, as shown in Figure 4, which illustrates the pooled effects of Cytoflavin on cognitive performance.

**Figure 4:**
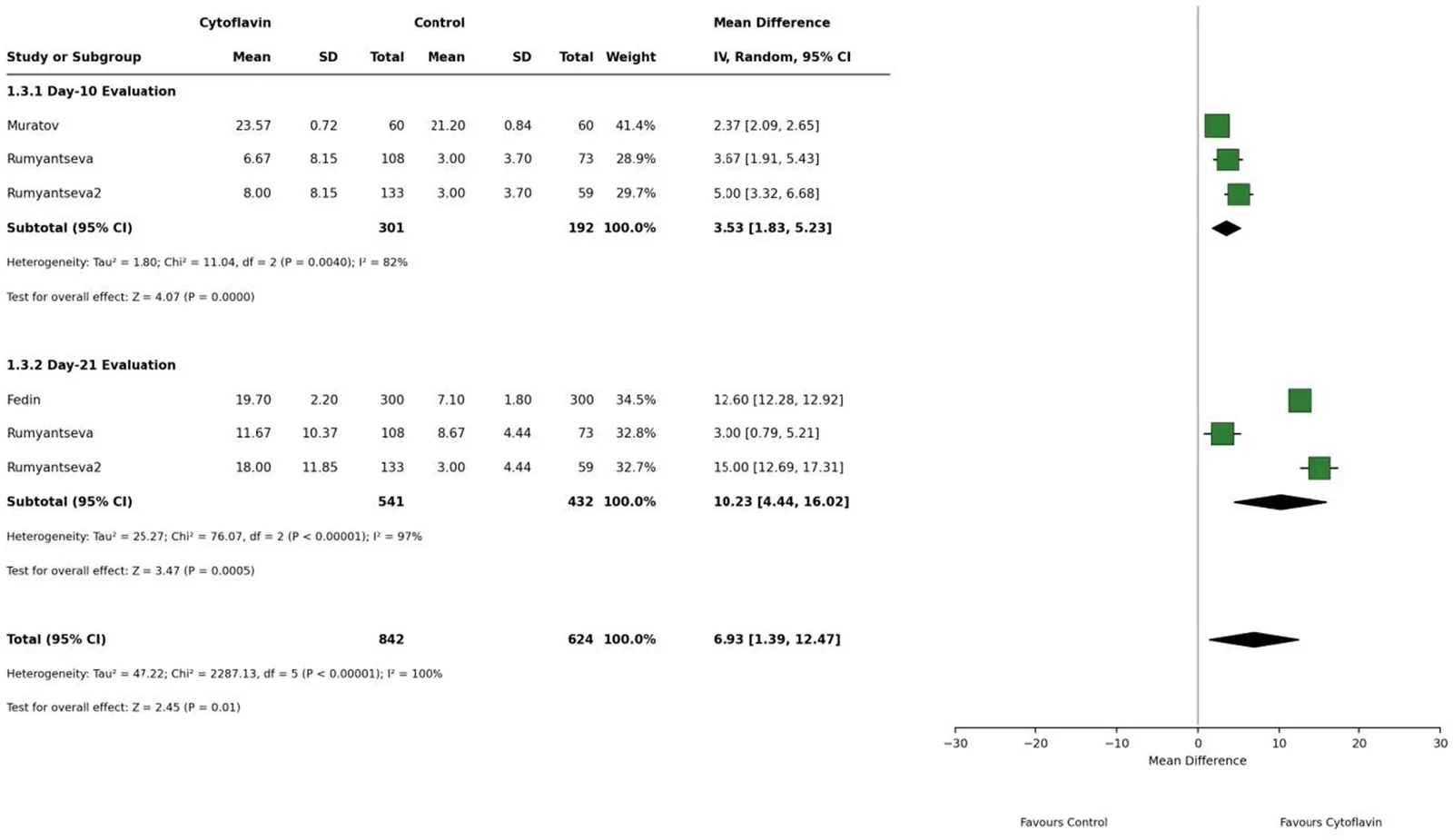
Forest plot of the meta-analysis assessing the effect of Cytoflavin vs. control on MMSE Outcomes at Day-10 and Day-21. The size of each square represents the weight of the study in the random-effects model, with horizontal lines showing the 95% confidence intervals. The diamond at the bottom indicates the pooled effect estimate and its 95% CI. The analyzed values reflect the post-treatment scores at the respective evaluation time points.

Publication bias assessment was re-verified for all outcomes after an initial data-entry duplication was identified between the MMSE and mRS outcomes; this prompted a full recomputation of Egger's and Begg's tests directly from the split-control-corrected, study-level effect sizes and standard errors for every outcome in this review (see NIHSS and Barthel Index above). For MMSE, recomputing Egger's regression test and Begg's rank correlation test directly from the extracted study-level effect sizes and standard errors (the same data underlying Figure 4) yielded Egger's *P* = 0.94 and Begg's *P* = 0.72, indicating no significant publication bias, consistent with our original conclusion. We note that, with only 6 comparisons contributing to this outcome across both time points, Egger's and Begg's tests have limited power to detect asymmetry (approximately 10 or more studies are generally recommended for reliable interpretation; Sterne et al., 2011), and these results should accordingly be read as exploratory. According to GRADE, the certainty of evidence was rated as low, primarily due to risk of bias and inconsistency.

#### 4.4.4 Modified Rankin Scale (mRS)

A total of 3 unique studies (4 dose-arm comparisons: Muratov et al., 2016; Sazonov et al., 2016, two dose arms; and Sazonov et al., 2016) were included in the meta-analysis for mRS. Although Rumyantseva et al. (2017) did not contribute to this outcome, Sazonov et al. (2016) contributed two Cytoflavin dose arms (10 mL/day and 20 mL/day) compared against a single shared control group; the shared control was split proportionally between the two dose-arm comparisons to correct for the same unit-of-analysis error described above. After this correction, 261 unique participants were included. As the Modified Rankin Scale is an ordinal measure, its use as a continuous outcome warrants comment: because the included studies reported mRS exclusively as continuous summary statistics (mean and standard deviation) rather than as dichotomized proportions, the mean difference was calculated to enable quantitative synthesis across studies, consistent with established practice for pooling ordinal stroke outcomes reported in this format. Since Day-10 was the only time point with consistent evaluation across all included studies, the analysis focused on this time point.

For Day-10, the pooled mean difference (MD) was 0.41 (95% CI: −0.74 to −0.09), indicating a significant improvement favoring Cytoflavin over control in reducing disability. Heterogeneity for Day-10 remained high (I^2^ = 97.5%), suggesting considerable variability in study results. The overall test for effect was significant (*P* = 0.01). The data for mRS were synthesized using random-effects models, with the results visually represented in the forest plot, as shown in Figure 5, which illustrates the pooled effects of Cytoflavin on global disability.

**Figure 5:**
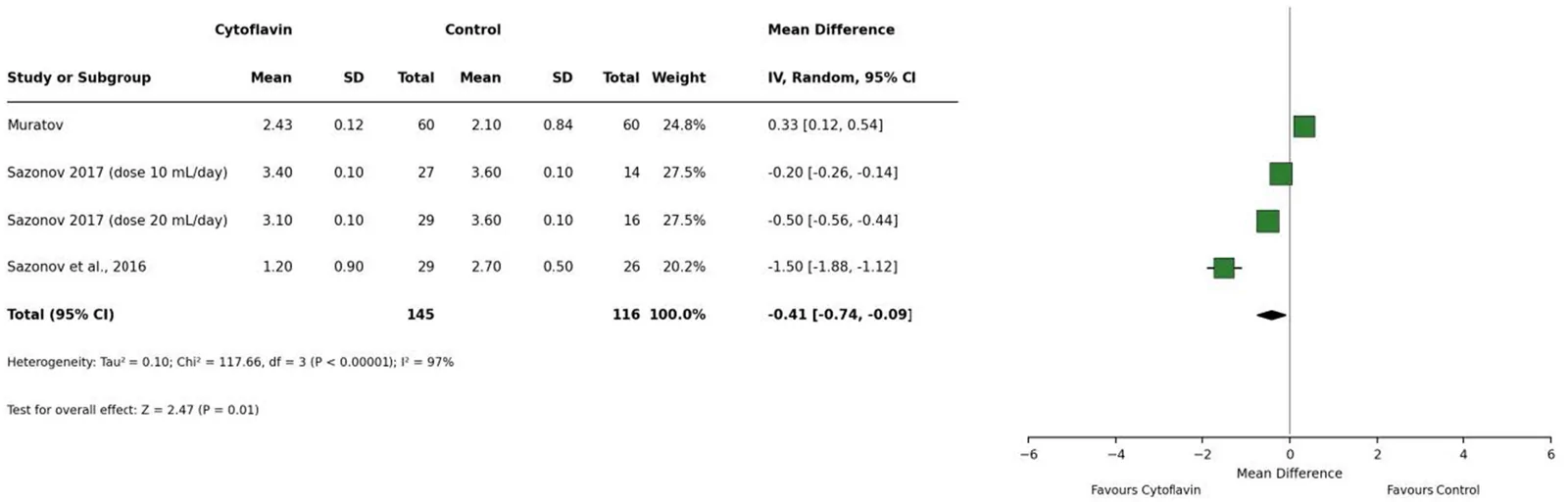
Forest plot of the meta-analysis assessing the effect of Cytoflavin vs. Control on mRS Outcomes at Day-10. The size of each square represents the weight of the study in the random-effects model, with horizontal lines showing the 95% confidence intervals. The diamond at the bottom indicates the pooled effect estimate and its 95% CI. The analyzed values reflect the post-treatment scores at the respective evaluation time points.

Publication bias assessment was likewise re-verified for the mRS outcome. Recomputing Egger's and Begg's tests directly from the study-level effect sizes and standard errors underlying Figure 5 yielded Egger's *P* = 0.97 and Begg's *P* = 1.00, indicating no significant publication bias and confirming our original conclusion. As with MMSE, this result is based on only 4 comparisons and should be interpreted as exploratory given the limited power of these tests with so few studies (Sterne et al., 2011). GRADE assessment rated the certainty of evidence as low, downgraded due to risk of bias and inconsistency.

#### 4.4.5 Infarct volume

A total of 2 studies were included in the meta-analysis for infarct volume (Table 1 lists a third study, Skvortsova et al. (2010), as also reporting this outcome, but its data do not contribute a row to the pooled analysis; see Strengths and Limitations). Rumyantseva et al. (2017) contributed two Cytoflavin dose arms (10 mL/day and 20 mL/day) at Day-21, compared against a single shared control group, which was split proportionally between the two dose-arm comparisons to correct for the same unit-of-analysis error described above. The analysis focused on two time points: Day-10 and Day-21 evaluations. For Day-10, 120 participants were included, and for Day-21, 373 unique participants were included after correction (compared with 505 participants prior to correction, which double-counted the shared control group).

For Day-10, the pooled mean difference (MD) was 0.17 (95% CI: 0.12 to 0.22), showing a slight increase in infarct volume in the Cytoflavin group compared to the control group. Heterogeneity for Day-10 was not applicable since only one study was included in the analysis. The overall test for effect was significant (*P* < 0.00001).

For Day-21, the pooled MD was −4.73 (95% CI: −11.41 to 1.95), suggesting a potential reduction in infarct volume favoring Cytoflavin, but the confidence interval includes zero, indicating some uncertainty in the effect. Heterogeneity for Day-21 was low (I^2^ = 0%), with the test for overall effect yielding *P* = 0.16.

Given the opposing direction of effect between evaluation time points, infarct volume outcomes are reported separately for Day-10 and Day-21 rather than as a single pooled estimate. At Day-10, based on a single available study, the mean difference was +0.17 cm^3^ (95% CI: 0.12 to 0.22), indicating a marginal increase in infarct volume; because this estimate derives from one study, it should be interpreted as preliminary rather than generalizable. At Day-21, drawing on two dose-arm comparisons from Rumyantseva et al. (2017) with the shared control split proportionally between them, the pooled mean difference was−4.73 cm^3^ (95% CI: −11.41 to 1.95), suggesting a reduction in infarct volume favoring Cytoflavin that did not reach statistical significance. The data for infarct volume were synthesized using random-effects models, with the results visually represented in the forest plot, as shown in Figure 6, which illustrates the treatment effect on infarct volume reduction at each evaluation time point.

**Figure 6:**
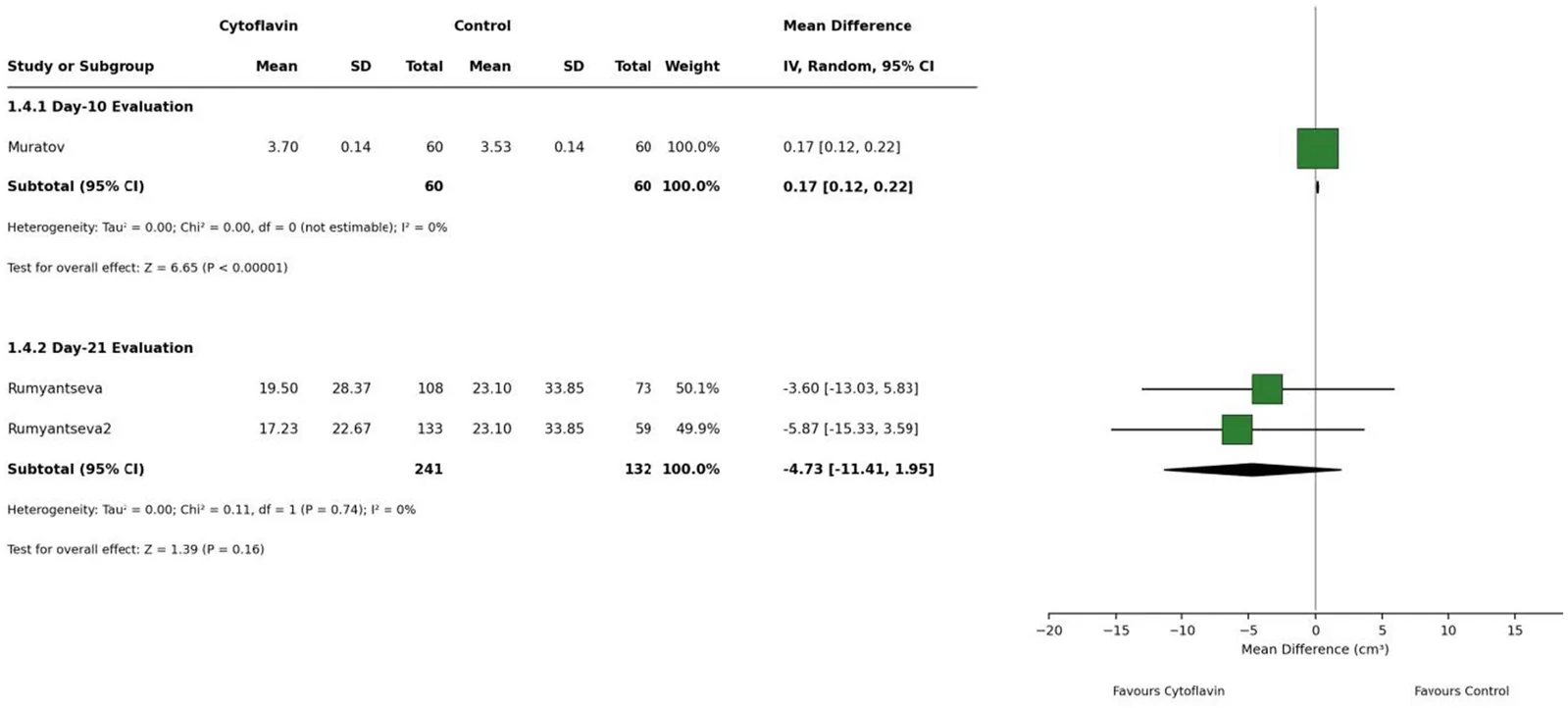
Forest plot of the meta-analysis assessing the effect of Cytoflavin vs. Control on infarct volume Outcomes at Day-10 and Day-21. The size of each square represents the weight of the study in the random-effects model, with horizontal lines showing the 95% confidence intervals. The diamond at the bottom indicates the pooled effect estimate and its 95% CI. The analyzed values reflect the post-treatment scores at the respective evaluation time points.

Publication bias assessment using Egger's (*P* = 0.392) and Begg's (*P* = 0.543) tests indicated no significant publication bias. According to the GRADE framework, the certainty of evidence was rated as very low, due to risk of bias, inconsistency, imprecision, and potential publication bias.

#### 4.4.6 Sensitivity Analysis

A sensitivity analysis was conducted by excluding observational studies (Sazonov et al., 2016 and Kovalenko et al., 2023) to assess the robustness of the meta-analysis exclusively on Randomized Controlled Trials (RCTs), using the unit-of-analysis-corrected (split-control) dataset described in Section 2.5. Results are reported numerically for each outcome and time point in which at least one observational study contributed: For NIHSS Day-10 (excluding Kovalenko et al., 2023), the pooled MD changed from −2.62 (95% CI: −3.35 to −1.88; k = 6, n = 779) with all studies to −2.78 (95% CI: −3.55 to −2.00; k = 5, n = 579) with RCTs only; the effect remained significant in both analyses (*P* < 0.0001). For NIHSS Day-21 (excluding Kovalenko et al., 2023), the pooled MD changed from −3.32 (95% CI: −6.53 to −0.11; k = 4, *n* = 1173; *P* = 0.04) with all studies to −4.45 (95% CI: −9.28 to 0.37; k = 3, n = 973; *P* = 0.07) with RCTs only; although the point estimate moved further in favor of Cytoflavin, statistical significance was not retained once the observational study was excluded, reflecting the loss of precision from a smaller RCT-only sample. For Barthel Index Day-10 (excluding Sazonov et al., 2016), the pooled MD changed from 14.42 (95% CI: 2.57 to 26.26; k = 6, *n* = 634; *P* = 0.02) with all studies to 12.34 (95% CI: −0.85 to 25.53; k = 5, *n* = 579; *P* = 0.07) with RCTs only, which was no longer statistically significant. For Barthel Index Day-21 (excluding Kovalenko et al., 2023), the pooled MD changed from 29.54 (95% CI: 2.09 to 56.98; k = 3, *n* = 573; *P* = 0.03) with all studies to 44.46 (95% CI: 39.57 to 49.34; k = 2, *n* = 373; *P* < 0.0001) with RCTs only, with the effect remaining significant and heterogeneity dropping sharply (I^2^ = 97.6% to I^2^ = 0%) once the null-effect observational study was removed. For mRS Day-10 (excluding Sazonov et al., 2016), the pooled MD changed from −0.41 (95% CI: −0.74 to −0.09; k = 4, n = 261; *P* = 0.01) with all studies to−0.14 (95% CI: −0.46 to 0.17; k = 3, n = 206; *P* = 0.37) with RCTs only, which was no longer statistically significant. The outcomes for MMSE and infarct volume were unaffected by this sensitivity analysis, as their pooled estimates were derived exclusively from RCTs with no observational studies contributing. Overall, three of the five sensitivity comparisons (NIHSS Day-21, Barthel Day-10, mRS Day-10) lost statistical significance when restricted to RCTs only, indicating that part of the evidence for these specific time points is driven by the inclusion of observational data and should be interpreted with corresponding caution; the remaining comparisons (NIHSS Day-10, Barthel Day-21) were robust to this exclusion.

## 5 Discussion

### 5.1 Summary of main findings

This meta-analysis demonstrated that Cytoflavin significantly improved several key outcomes in ischemic stroke, including neurological function (measured by NIHSS scores), functional independence (Barthel Index), cognitive performance (MMSE scores), and global disability (mRS scores). Its effect on infarct volume appeared time-dependent, with a more favorable trend observed at Day-21 than at Day-10, and both the heterogeneity across studies and the geographic concentration of the evidence base (almost entirely from Russia and Uzbekistan) warrant caution in interpreting these findings.

### 5.2 Interpretation of results

Cytoflavin's effects on neurological recovery, functional independence, and cognitive performance are evident, with improvements in NIHSS, Barthel Index, and MMSE. Its effect on infarct volume followed a time-dependent pattern, with a more favorable trend at the later evaluation point. These findings suggest that Cytoflavin has the potential to improve multiple aspects of post-stroke recovery.

The effect of Cytoflavin on infarct volume appears to be time-dependent. At Day-21, the pooled estimate (MD = −4.73 cm^3^, 95% CI:−11.41 to 1.95) points toward a reduction in infarct volume, although the confidence interval does not exclude the null. The Day-10 estimate (MD = +0.17 cm^3^) is derived from a single study and is accordingly limited in generalizability. Taken together, these findings suggest that Cytoflavin's effect on infarct volume may take time to manifest and warrants confirmation in studies with multiple Day-10 measurements and longer follow-up.

Cytoflavin may act through its multifactorial pharmacodynamics, including enhanced mitochondrial respiration, reduced oxidative stress, and modulation of neuroplasticity. The notable effect on MMSE scores points to Cytoflavin's potential neuroprotective and neuroreparative actions in post-stroke cognitive impairment. The magnitude of cognitive improvement was also significantly larger at Day-21 than at Day-10 (subgroup difference *P* = 0.03), suggesting that Cytoflavin's cognitive benefits may accumulate with continued treatment duration rather than appearing immediately post-stroke.

### 5.3 Heterogeneity and bias analysis

The high heterogeneity observed (I^2^ ranging from 0% (Infarct Volume, Day-21) to 100% (Barthel Index, Total), with MMSE Total also very high (I^2^ = 99.8%), across outcomes, with most individual comparisons in the high range (approximately 82%-100%)) may be attributed to differences in study design, dosage regimens, and timing of Cytoflavin initiation. Variability in control groups (standard care vs. alternative neuroprotectors, such as ascorbic acid or piracetam) may also have contributed to the observed heterogeneity across the studies. This heterogeneity persisted after correcting the unit-of-analysis error described in Section 2.5 (Data Synthesis), indicating that it reflects genuine clinical and methodological diversity across the included studies rather than being primarily an artifact of the double-counted control groups. Although publication bias was assessed using Egger's and Begg's tests, no significant bias was found across major outcomes, indicating that bias is unlikely to substantially affect the overall conclusions. As prespecified in Section 2.5, meta-regression to explore covariates such as Cytoflavin dosage or baseline NIHSS severity was not performed, since our limited number of included trials per clinical outcome did not meet the minimum of 10 studies per covariate required for a sufficiently powered analysis. Therefore, we addressed this variability primarily through subgroup analyses (Day-10 versus Day-21 evaluation points) and sensitivity analyses isolating study designs.

A related point concerns why studies with markedly different sample sizes do not proportionally dominate the pooled estimates in the forest plots (Figures 2–6); for instance, in the NIHSS Day-21 subgroup (Figure 2), Fedin et al. (2005, *n* = 600) and Camandola and Mattson (2017, n = 200) receive comparable weight (25.8% and 25.0%, respectively) despite a three-fold difference in participant numbers. Under a fixed-effect model, the larger study would indeed be weighted more heavily, driven by its smaller within-study variance. However, all outcomes in this review were pooled using the DerSimonian-Laird random-effects model (Section 2.5), in which each study's weight is the inverse of the sum of its within-study variance and the between-study variance (Tau^2^): Weight = 1 / (v_i_ + Tau^2^). Because heterogeneity was very high across outcomes (I^2^ up to 100%, corresponding to a large Tau^2^), the between-study variance dominates this denominator and tends to equalize weights across studies irrespective of sample size. This is an expected mathematical property of random-effects pooling under substantial heterogeneity rather than an analytic error, and it means the pooled estimates should be read as an average effect across heterogeneous study contexts rather than a precision-weighted estimate driven by the largest trials.

### 5.4 Comparison with previous studies

These findings align with previous studies, such as those by Kovalenko et al. (2023) and Rumyantseva et al. (2017), who reported improvements in neurological recovery and functional outcomes with Cytoflavin. Our findings on infarct volume add a temporal dimension to this literature, suggesting that treatment effects on infarct size may become more apparent at later evaluation points (Rumyantseva et al., 2017; Kovalenko et al., 2023).

Sazonov et al. (2017) suggested a partial dose–response relationship: the 20 mL/day arm showed numerically greater improvement than the 10 mL/day arm for NIHSS and mRS, whereas the reverse pattern was observed for the Barthel Index, indicating that the relationship between dose and outcome was not consistent across all measures. While our analysis did not directly assess dose-response effects, higher doses or longer treatment durations may lead to more significant outcomes, particularly in terms of infarct volume reduction, which warrants further investigation (Sazonov et al., 2017).

In patients with metabolic syndrome, Muratov et al. (2016) found a 35-day regimen of IV followed by oral Cytoflavin yielded cognitive and functional improvements. Our infarct volume findings complement this by pointing to a similar time-course, with the more pronounced effect emerging at the later evaluation point (Muratov et al., 2016).

While the mechanisms for these findings are supported by preclinical data, including Cytoflavin's role in reducing oxidative stress and modulating mitochondrial function, further molecular and clinical studies are needed to validate its neuroprotective pathways in human stroke. Our study indicates that Cytoflavin confers significant benefits for functional and cognitive recovery, with its effect on infarct volume emerging more clearly at later evaluation points, a pattern that merits further characterization in future trials with longer follow-up (McClave et al., 2019; Camandola and Mattson, 2017).

These findings align with the growing body of evidence emphasizing the importance of early neuroprotection in AIS (Saver, 2006; Ginsberg, 2008; Patel and McMullen, 2017). Current stroke guidelines support reperfusion therapy but highlight the need for adjunctive neuroprotective strategies (Powers et al., 2019).

Cytoflavin promotes improvement across multiple brain domains (neurological, cognitive, and autonomic) by supporting recovery of higher cortical functions such as memory, attention, speech, and executive function (Lautrup et al., 2019; Ying, 2008; Xiao et al., 2018; Ashoori and Saedisomeolia, 2014). These improvements are particularly relevant for post-stroke independence and social reintegration (Poltavskaya et al., 2020; Muratov et al., 2016). Although most studies report favorable outcomes, the magnitude of benefit varies, with more pronounced effects observed in patients with comorbid conditions such as metabolic syndrome (Muratov et al., 2016).

In a randomized controlled trial by Poltavskaya et al. (2020), patients receiving Cytoflavin showed greater improvements in MMSE (5.8% vs. 1.6%) and NIHSS (17.6% vs. 10.8%) compared to control (Poltavskaya et al., 2020). A study by Karakulova et al. (2018) demonstrated improved cognitive functions and increased levels of brain-derived neurotrophic factor (BDNF), suggesting a role in promoting neuroplasticity and cerebral autoregulation (Karakulova et al., 2018).

Additionally, Cytoflavin has been reported to support recovery of central nervous system regulatory processes, particularly respiration and consciousness, in postoperative contexts. Evidence from clinical studies indicates it may reduce arousal time after general anesthesia, especially in patients experiencing prolonged postanesthetic clouding of consciousness (Novikov et al., 2017; Ovezov et al., 2010).

A clinical comparison against another antioxidant agent offers further mechanistic context. A randomized trial by Kataeva et al. (2022) compared Cytoflavin with ethylmethylhydroxypyridine succinate in patients with chronic brain ischemia and found that, while both agents produced comparable reductions in oxidative stress and overall neurocognitive deficit, Cytoflavin was specifically superior in enhancing perceptual selectivity and attention, whereas ethylmethylhydroxypyridine succinate was more effective at reducing reaction time to moving stimuli, suggesting the two agents may act through partly distinct neurophysiological pathways despite their shared antioxidant profile (Kataeva et al., 2022).

### 5.5 Strengths and limitations

This meta-analysis' strengths include the rigorous selection of studies and comprehensive assessment of multiple clinical endpoints. However, some studies had methodological flaws, and the heterogeneity observed limits the certainty of pooled estimates. The majority of studies were conducted in Russia, which may affect external validity, and few studies reported long-term outcomes beyond 3 months. Furthermore, a notable limitation regarding the external validity of our findings is that all included primary studies were strictly conducted within Russia and Uzbekistan, where Cytoflavin is regionally integrated into standard stroke protocols. Consequently, the efficacy and safety profile observed in this meta-analysis may not directly generalize to other global healthcare systems. Differences in ethnic demographics, genetic predispositions to stroke pathways, and variations in baseline acute stroke management protocols across different regions necessitate cautious international translation of these results until broader multi-regional trials are available. Additionally, this review restricted eligibility to studies published in English or Russian; relevant studies published in other languages may have been missed, which represents a further potential source of selection bias. A further methodological limitation concerns the modified Rankin Scale (mRS), an ordinal measure of global disability that we analyzed using mean difference because the included studies reported it only as continuous summary statistics rather than as dichotomized proportions (see Section 4.4.4); an ordinal-logit approach, or pooling a prespecified dichotomy (e.g., mRS 0-2 vs. 3-6), would have been methodologically preferable had the primary data been available in that format, and this constraint should be considered when interpreting the mRS findings, which in any case carry low certainty of evidence per GRADE. A further limitation concerns the choice of appraisal tool for the observational studies: the Newcastle-Ottawa Scale (NOS) was used rather than ROBINS-I, which is increasingly recommended by Cochrane guidance for non-randomized studies of interventions; this choice was made because the included observational studies did not consistently report sufficient confounder-level detail for a full ROBINS-I assessment (see Risk of Bias and Quality Assessment), and future reappraisal with ROBINS-I, should fuller study-level data become available, may yield a more granular picture of bias in these studies. We also note that Table 1 (Study Characteristics) lists three additional studies, Skvortsova et al. (2010); Polysan (2003), and Kovalchuk et al. (2018), as having reported one or more of this review's outcomes of interest (NIHSS, Barthel Index, and/or MMSE), yet these studies do not contribute a row to the corresponding forest plots (Figures 2, 3, 4, and 6, as applicable to each study-outcome pairing). This is because their outcome data, as extracted, were not reported in a mean-and-standard-deviation format compatible with the mean-difference pooling method used throughout this review (e.g., some were reported as percentage change or categorical improvement rather than as continuous summary statistics); as such, they could not be quantitatively synthesized alongside the other included studies. We further note that Table 1 lists Fedin et al. (2005) as reporting Barthel Index, yet this study likewise does not appear in the Barthel Index forest plot (Figure 3); as we could not locate a corresponding mean/SD extraction for this study-outcome pairing in our data files, we flag this specifically for verification against the original data-extraction records, alongside the studies named above, before final submission to confirm no extractable data were inadvertently omitted. Finally, we note that Egger's and Begg's tests for publication bias were applied to each outcome despite most outcomes comprising fewer than 10 constituent studies or comparisons (ranging from k = 4 for mRS to k = 10 for NIHSS, the latter at the threshold itself); current methodological guidance suggests these regression-based tests require approximately 10 or more studies to yield a reliably powered estimate of funnel-plot asymmetry. All publication-bias results reported in this review, not only those flagged for the MMSE and mRS outcomes above, should therefore be interpreted as exploratory and interpreted with corresponding caution rather than as definitive evidence of an absence of publication bias.

### 5.6 Clinical implications and future directions

Cytoflavin shows potential for integration into standard post-stroke rehabilitation protocols to improve neurological function, cognitive restoration, and functional independence. However, high heterogeneity indicates the need for large-scale, multicenter randomized controlled trials (RCTs) with standardized protocols to confirm these benefits.

Further high-quality, multicenter RCTs are needed to confirm efficacy across diverse populations and explore long-term outcomes. Comparisons with other neuroprotective agents such as edaravone, N-acetylcysteine, and uric acid would be informative. Additionally, studies incorporating biomarkers of oxidative stress, mitochondrial function, and inflammation are warranted to substantiate Cytoflavin's mechanisms of action.

## 6 Conclusion

This meta-analysis demonstrates that Cytoflavin shows promising neuroprotective effects in acute ischemic stroke, with significant improvements in neurological recovery (NIHSS) and functional independence (Barthel Index), both rated as moderate certainty of evidence, alongside improvements in cognitive performance (MMSE) and global disability (mRS), both rated as low certainty of evidence, and a time-dependent trend toward reduced infarct volume (very low certainty of evidence) that was more pronounced at later evaluation. The mRS finding in particular should be interpreted with caution, as it was derived using a mean-difference approach to an inherently ordinal scale, as discussed in the Limitations. Given the substantial inter-study heterogeneity (I^2^ up to 100% for several outcomes) and the fact that the entire evidence base originates from studies conducted in Russia and Uzbekistan, these findings should be interpreted as a regional signal of promise rather than established evidence of efficacy, and the real-world usefulness and effectiveness of Cytoflavin must be independently verified by multi-regional, high-quality randomized controlled trials conducted by other research teams before it can be considered for integration into standard global stroke protocols.

## Data Availability

The datasets presented in this article are not readily available because no new datasets were generated or analyzed in this study. All data used in this systematic review and meta-analysis were derived from previously published studies and are available in the public domain. Therefore, no restrictions apply. Requests to access the datasets should be directed to dodiktugasworo152314@gmail.com.
